# Interleukin-37 suppresses tumor growth through inhibition of angiogenesis in non-small cell lung cancer

**DOI:** 10.1186/s13046-016-0293-3

**Published:** 2016-01-20

**Authors:** Guanqun Ge, Aiqin Wang, Jingyue Yang, Yan Chen, Jin Yang, Yize Li, Yan Xue

**Affiliations:** Department of Surgical Oncology, The First Affiliated Hospital of Xi’an Jiaotong University, 277, Yantaxi Road, Xi’an, 710061 Shaanxi China; Department of Neurosurgery, The Ninth Hospital of Xian, Xi’an, 710054 China; Department of Oncology, Xijing Hospital, The Fourth Military Medical University, No.15 Changle West Road, Xi’an, Shannxi 710032 China

**Keywords:** Interleukin-37, Non-small cell lung cancer, Tumor angiogenesis, Microvessel density

## Abstract

**Background:**

Interleukin-37 (IL-37), a newly identified member of the IL-1 family, has been known to play an immunosuppressive role in a variety of inflammatory disorders, but whether it participates in the regulation of pathogenesis of non-small cell lung cancer (NSCLC) has not been investigated.

**Methods:**

Real-time polymerase chain reaction (PCR), western blotting, and immunohistochemical staining were employed to detect IL-37 expression in NSCLC tissues and corresponding adjacent tissues. The correlations between IL-37 expression and clinicopathological characteristics, prognosis were analyzed. Stable clone with overexpression of IL-37 was generated in H1299 cell lines. Cell growth, cell cycle and cell apoptosis assays were carried out for detecting proliferation and apoptosis of H1299 cells. The effects of IL-37 on NSCLC progression in vivo was performed in a xenografted lung tumor model in nude mice. The concentrations of IL-37 and VEGF in the s growth medium supernatants were quantified by ELISA. The antiangiogenic effect of IL-37 on HUVEC was measured by tube formation assay.

**Results:**

IL-37 mRNA and protein expressions were significantly decreased in NSCLC tissues, and decreased intratumoral IL-37 expression was significantly associated with tumor state, TNM stage and poor prognosis in NSCLC patients. In addition, intratumoral IL-37 expression was an independent prognostic factors for Overall survival (hazard ratio = 2.047; *P* = 0.011). Overexpression of IL-37 exerted no direct effect on cell proliferation and apoptosis of H1299 lung cancer cells in vitro, but significantly inhibited tumor growth in a H1299 xenograft model in vivo. Furthermore, there was no significant change in immune cell infiltration in IL-37 over-expressing tumors; instead, we found decreased microvessel density (MVD) and VEGF levels in IL-37-expressing tumors. Additional studies showed IL-37 could directly inhibit HUVEC cells growth and capillary structure formation. Finally, we found that decreased IL-37 expression was associated with high MVD in NSCLC patients.

**Conclusions:**

Our findings demonstrate a protective role for IL-37 in lung cancer development, possibly through inhibiting tumor angiogenesis. IL-37 could serve as a promising therapeutic target for NSCLC.

**Electronic supplementary material:**

The online version of this article (doi:10.1186/s13046-016-0293-3) contains supplementary material, which is available to authorized users.

## Background

Non-small cell lung cancer (NSCLC) accounted for approximately 80–85 % of all lung cancers, it is currently the most common cause of tumor-related mortality in both men and women around the world [1]. Despite advances in early detection, radical surgical resection, and multimodal therapeutic modalities over the recent decades, the long-term survival remains poor due to the high rate of recurrence and metastasis [1]. Therefore, there is an urgent need to identify novel biomarkers that will help select the patients with high chance of lung cancer recurrence and provide better prognosis and individualization treatment.

Interleukin-37 (IL-37, formerly named IL-1 F7, IL-37b), which was reported by several independent groups in the year 2000, is a newly identified member of the interleukin-1 (IL-1) family [2–4]. There are five different splice variants of IL-37 termed IL-37a-e, IL-37a, b, and d are recognized as the functional forms of IL-37 [2–5]. IL-37b is the largest isoform and was detected in several human tissues, including lung and testis, and in colon tumors and human cell lines, such as THP-1, U937 and A431 [6, 7]. IL-37 has been identified as a natural suppressor of innate immunity and inflammatory responses [8, 9]. Recently, three groups have demonstrated that IL-37 requires the receptors IL-18Rα and IL-1R8 to carry out its multifaceted anti-inflammatory program upon innate signal transduction [10–12]. It has been demonstrated that IL-37 can be induced by several toll-like receptor (TLR) ligands and pro-inflammatory cytokines such as IL-1β, TNF-α, IFN-γ in PBMCs [13, 14]. However, over-expressed human IL-37 inhibited the TLR-induced pro-inflammatory cytokines in mouse macrophage RAW cell line, human monocytic cell line THP-1 and epithelial cell line A549 [14]. IL-37 transgenic mice can markedly reduce clinical manifestations of DSS colitis [15], ischemia-reperfusion injury [16–18], obesity-induced inflammation [19], LPS-induced shock and psoriasis [20], and ameliorate their inflammatory cytokine productions. In clinical practice, higher levels of serum IL-37 have been detected in patients with autoimmune diseases, such as rheumatoid arthritis [21], inflammatory bowel disease [22], systemic lupus erythematosus [23], Graves’ Disease [24] and Guillain-Barré Syndrome [25].

Recently, three previous studies demonstrated that IL-37 play a protective role in tumor progression. Gao et al. found that treatment of an established MCA205 mouse fibrosarcoma model by single intratumoral injection of Ad-IL-37 resulted in significant growth suppression, and the antitumor effect of IL-37 was dependent on T cells and B cells [7]. Zhao et al. reported that IL-37 mediated anti-tumor immune responses through recruiting NK cells to tumor microenvironment in hepatocellular carcinoma (HCC) [26]. Recent study by Wang found that IL-37 suppressed cell proliferation and invasion of human cervical cancer (CC) through inhibiting STAT3 signaling [27]. Despite this knowledge, the biological role of IL-37 in tumor development remains largely unknown.

To address the function of IL-37 in lung cancer, we first evaluated IL-37 expression in the human NSCLC tissues; IL-37 function was assessed in vitro in IL-37 over-expressing lung cancer cell lines, and in vivo in a xenografted lung tumor model. Our results show that IL-37 plays an inhibitory role in lung cancer development, possibly through inhibiting tumor angiogenesis.

## Methods

### Patients and specimens

A total of 182 patients who underwent surgery for histologically verified NSCLC at the Xijing Hospital between 2007 and 2010 were enrolled in this study. The median age of the patients was 54.7 years (range 27–76 years). None of them received any preoperative anticancer treatment prior to sample collection. This study was approved by the local ethics committee and written informed consent was obtained from each patient. All 182 specimens were reevaluated with respect to their histological types, differentiation status, smoking status, and tumor TNM stages. Tumor stages were determined by TNM classification according to the 2002 International Union against Cancer guidelines. The histological diagnosis and grade of differentiation of the tumors were defined by evaluation of the hematoxylin and eosin-stained tissue sections, according to the World Health Organization guidelines of classification (2004). Overall survival (OS) was defined as the interval between surgery and death. Patients in whom recurrence was not detected were censored on the date of death or the last follow-up. The clinicopathologic characteristics of these 182 patients are listed in Table 1. Every patient specimen included two matched pairs, namely, NSCLC tissues and adjacent normal lung tissues (≥5 cm away from the tumor). Tissues were collected within 1 h after surgery. For each specimen, half were immediately flash-frozen in liquid nitrogen and then frozen at −80 °C until RNA and protein extraction was performed, the remainder was fixed with formalin for immunohistochemistry.

**Table 1:** Association between intratumoral IL-37 expression and clinicopathological characteristics of 182 NSCLC patients

Characteristics	Case (182)	IL-37 expression		*P* value
		High (76)	Low (106)	
Age (years)				
≤60	102	44	58	0.670
>60	80	32	48	
Gender				
Male	129	51	78	0.343
Female	53	25	28	
Histological type				
Adenocarcinoma	86	37	49	0.223
Squamous cell carcinoma	66	23	43	
others	30	16	14	
Smoking status				
Smoker	112	46	66	0.812
Non-smoker	70	30	40	
Differentiation				
Well-moderate	40	17	23	0.914
Poor	142	59	83	
Tumor status				
T1-T2	121	58	63	0.017
T3-T4	61	18	43	
TNM stage				
I-II	122	62	60	0.000
III	60	14	46	

### Immunohistochemical staining for IL-37 and CD34

Surgically excised tumor specimens and were fixed with 10 % neutral formalin and embedded in paraffin, and 4-μm-thick sections were cut for immunohistochemical analysis. Sections were dewaxed in xylene and rehydrated through graded alcohols.

For IL-37 immunostaining, a microwave-based antigen retrieval process was employed with EDTA buffer, pH8.0, for 30 min. After the sections had been cooled, endogenous peroxidase was inhibited with 3 % hydrogen peroxide for 10 min at room temperature. Non-specific binding was blocked with fetal calf serum for 15 min before incubation of the sections with mouse anti-human IL-37 antibody (ab57187, 1:1000 dilution, Abcam, Cambridge, MA, USA) at 4 °C overnight. As a negative control, sections were incubated with normal mouse IgG. After being incubated with the primary antibodies, the sections were then incubated with horseradish peroxidase (HRP)-labeled anti-mouse IgG at 37 °C for 30 min, followed by visualization with 3, 3-diaminobenzidine (DAB) and counterstaining with Mayer’s hematoxylin. Desired color reaction was observed when monitored with the microscope.

Immunostaining for CD34 was performed as previously described [28]. Briefly, after sections were dewaxed and rehydrated, the antigen retrieval and inhibition of the endogenous peroxidase processes were performed. The sections were then incubated with mouse anti-human CD34 monoclonal antibody (1:200 dilution; Zhongshan Golden Bridge Biotech., Beijing) for 30 min at 37 °C, followed by incubation with EnVision Detection (K5007, DAKO, Denmark) for 30 min at room temperature.

### Immunohistochemistry evaluation

Analysis was performed by two independent pathologists in a blinded manner. Tissue sections were screened at low power field (100×) and five most representative fields were selected at high power field (400×) with Leica DM IRB inverted research microscope (Leica Microsystems, Wetzlar, Germany). IL-37 density was quantified according to the percentage of positively stained cells and the staining intensity. The staining extent was scored from 0 to 3 based on the percentage of positive cells (0, < 5 %; 1, 5 %-25 %; 2, 25 %-50 %; 3, >50 %). The intensity of staining was classified as follows: 0 point, no staining; 1 point, weak staining (light yellow); 2 points, moderate staining (brown); and 3 points, strong staining (yellowish brown). The final score of IL-37 expression was calculated as the percentage positive score × the staining intensity score, graded as 0–1 for negative, + for 2–3 points, ++for 4–6 points, and +++ for 7–9 points. When there were discrepancies between the two pathologists, the average score was used. Tumors with final staining score ≥4 were defined as high IL-37 expression group.

For intratumoral MVD quantification, microvessels were recorded by counting CD34-positive immunostainedendothelial cells according to the international consensus on the methodology and criteria of evaluation of angiogenesis quantification in solid tumors [29]. Briefly, the immunostained sections were initially scanned at low power (100× magnification) to identify the “hot spots,” which are the areas with the highest vascularity. Subsequently, counts of the stained microvessels were performed on three consecutive high-power (200× magnification) fields within the hot spots. Any yellow-brown immunostained endothelial cell or endothelial cell cluster that was clearly separate from adjacent microvessels was considered as a single countable microvessel. The average of three 200× field microvessel counts was recorded as the value of MVD.

### Cell culture

The H1299 and A549 human lung cancer cell lines and human umbilical vein endothelial cells (HUVEC) were obtained from the American Type Culture Collection (ATCC; Rockville, MD, USA) and cultured in DMEM (GIBCO, Shanghai, China) supplemented with 10 % FBS. Human IL-37 (NM_014439) cDNA open reading frame (Origene Technologies, Beijing, China) were cloned into pcDNA3.1 vector. The IL-37 plasmids or pcDNA3.1 vector were transfected into cells using Lipofectamine^®^ 2000. 48 h after transfection, transfectants were selected in culture medium supplemented with 600 μg/ml G418. G418-resistant monoclones were picked and expanded in the selection medium.

### Quantitative RT-PCR

RNA was extracted using Trizol (Invitrogen, Carlsbad, CA, USA) method and reverse transcripted to cDNA using RT Kit (Invitrogen). Quantitative PCR analyses were performed on ABI 7500 fast real-time PCR system (Applied Biosystems, Foster City, CA, USA) using the SYBR Green PCR Master Mix (Applied Biosystems). Primer sequences were as follows: forward: 5'-GATCACAAAGTACTGGTCCTGG-3', reverse: 5’-TCCTTTATCCTTGTCACAGTAG-3’ for IL-37b; forward: 5'- GTGGACATCCGCAAAGAC-3', and reverse 5'- AAAGGGTGTAACGCAACTA -3' for β-actin. The expression level of IL-37 was normalized to the expression of the housekeeping gene β-actin using the comparative threshold cycle (2^-ΔΔCt^ ) method.

### Western blotting

Total protein from tumor tissues and cultured cells were lysed in RIPA buffer with protease inhibitor (Beyotime, Shanghai, China). The protein was quantified using a BCA assay kit (Beyotime, Shanghai, China). A total of 20 μg of total protein were separated by 10 % SDS-PAGE, transferred onto polyvinylidene fluoride membranes, and then reacted with primary antibodies against IL-37 (24 kDa, ab101376, 1:500), and β-actin (42 kDa, ab119716, 1:1000) (all from abcam, Cambridge, UK). After being extensively washed with PBS containing 0.1 % Triton X-100, the membranes were incubated with alkaline phosphatase-conjugated goat anti-rabbit antibody for 30 min at room temperature. The bands were visualized using 1-step TM NBT/BCIP reagents (Thermo Fisher Scientific, Rockford, IL) and detected by an Alpha Imager (Alpha Innotech, San Leandro, CA).

### ELISA

H1299, H1299-Mock, and two clones of IL-37-transfected H1299 cells with the same beginning numbers were cultured with same volume of medium and the growth medium supernatant were collected when cells reached 90 % confluence. The concentrations of secreted IL-37 and VEGF in the growth medium supernatants were quantified by using a commercial human IL-37 ELISA kit (AdipoGen AG, Liestal, Switzerland) and VEGF Kit (R&D Systems, Inc., Minneapolis, MN, USA) according to the manufacturer's protocol. All samples were assayed in duplicate.

### Cell viability assay

Cell viability was evaluated using CCK-8 (Beyotime, Shagnhai, China) according to manufacturer’s instructions. Briefly, two clones of IL-37-transfected H1299 cells; mock-transfected and wild-type H1299 cells were seeded into 96-well plates at 5 × 10^3^ cells per well and cultured for indicated time points. 10 μl of CCK-8 solution was added into the culture medium in each well. After 1 hour incubation, OD values were read using a microplate reader (Bio-Tek Company, Winooski, VT, USA) at the 450-nm wavelength. Each time point was repeated in three wells and the experiment was independently performed for three times. The effect of IL-37 on the proliferation of HUVEC cells was also determined by CCK-8 assay. Briefly, the HUVEC cells were seeded into 96-well plates at 1 × 10^4^ cells per well and incubated at 37 °C. After 24-hr incubation, they were treated with medium containing 50 % (V/V) IL-37-expressing supernatant from IL-37-transfected H1299 cells (Clone 2) for the indicated time periods (0–4 days). The medium containing 50 % (V/V) supernatant from mock-transfected H1299 cells was used as control. After treatments for different time periods, the HUVEC cells’ growth was examined as described above. The experiment was repeated at least three times.

### Cell cycle assay

Cell cycle was determined using propidium iodide (PI) staining by flow cytometry. Cells with the same beginning numbers (1 × 10^6^) were cultured at 37 °C for two days, the cells were harvested and washed in cold PBS, then fixed in 70 % cold alcohol for 6 hr at 4 °C, then washed in cold PBS, and stained with PI solution at 4 °C in the dark for 30 min. The cells were analyzed by flow cytometry (BD FACSCalibur, BD Bioscience, San Diego, CA, USA), data were analyzed using Flowjo software (FlowJo, Ashland, OR, USA). The experiment was repeated three times.

### Cell apoptosis assay

Cell apoptosis was evaluated by flow cytometry using an Annexin V-FITC Apoptosis Detection Kit (KeyGen Biotech Co. Roche, Nanjing, China). Briefly, cells were seeded into 24-well plates at 1 × 10^5^ cells per well and cultured in 0.1 % FBS medium for 48 h. Then the cells were detached by trypsinization, washed twice in PBS (2000 rpm, 5 min; Allegra X-12R centrifuge; Beckman Coulter, USA), and resuspended in 500 μL binding buffer. A volume of 5 μL Annexin V-FITC and 5 μL propidium iodide was added and mixed gently, and the cells were stained in the dark for 10 min at room temperature. The cells were analyzed immediately by flow cytometry (BD FACSCalibur, BD Bioscience, San Diego, CA, USA) and analyzed using Flowjo software (FlowJo, Ashland, OR, USA). The experiment was repeated three times.

### Animal study

Female 6–8 weeks old BALB/c nu/nu mice (Charles River Laboratories, Beijing, China) were housed in specific pathogen-free conditions. The study was approved by the Research Ethics Committee of The Fourth Military Medical University. For evaluation of the tumor growth in vivo, 5 × 10^6^ cells were suspended in 200 μl PBS and injected subcutaneously into the flank region of nude mice. Tumor growth was monitored every week and tumors were measured with fine digital calipers and tumor volume was calculated by the following formula: tumor volume =0.5 × width^2^ × length. Tumor-bearing mice were sacrificed 4 weeks after tumor inoculation and the tumors were removed, weighed, and separated into two equal parts: one part was used for FACS analysis, the other fixed in 10 % neutral formalin for immunostaining of CD34.

### FACS analysis of tumor infiltrating lymphocytes

Fresh removed tumor tissues were cut into small pieces, and separated into single cells using cell dissociation solution (100 U/ml Collagenase type IV and 100 μg/ml DNase in RPMI + 10 % FBS). Tumor infiltrating lymphocytes (TILs) were separated on a 40 %/70 % Percoll (GE Healthcare, Piscataway, NJ) gradient. Single cells were collected and stained with antibodies against CD11c, CD11b, Gr-1 and DX-5 (BioLegend, San Diego, CA, USA). Data were acquired on a FACS Calibur (BD Bioscience, San Diego, CA, USA) and analyzed using Flowjo software (FlowJo, Ashland, OR, USA).

### Tube formation assay

HUVEC cells were pretreated with medium containing 50 % (V/V) IL-37-expressing supernatant from IL-37-transfected H1299 cells (Clone 2) for 4 h. The medium containing 50 % (V/V) supernatant from mock-transfected H1299 cells was used as control. Then the cells were plated onto the layer of Matrigel (BD Bioscience, San Diego, CA, USA) at a density of 1 × 10^4^ cells/well. After 24 h, tubular structures were quantified and photographed under a microscope. The number of formed capillary tubes was manually counted.

### Statistical analysis

Statistical analyses were performed using the SPSS-PC package (version 19.0; SPSS, Chicago, IL, USA). The Chi-square test was used to analyze the association between IL-37 expression and various clinicopathologic factors. Spearman correlation test was used to evaluate the association between IL-37 mRNA levels and its protein levels. The Kaplan-Meier method with the log-rank test was used to calculate survival rates and differences in survival curves. A multivariate Cox proportional hazards regression model was employed to identify independent prognostic factors. A student’s t-test was performed to analyze differences between two groups. *P* < 0.05 was considered statistically significant.

## Results

### Decreased IL-37 expression is associated with poor prognosis in NSCLC patients

**Fig. 1:**
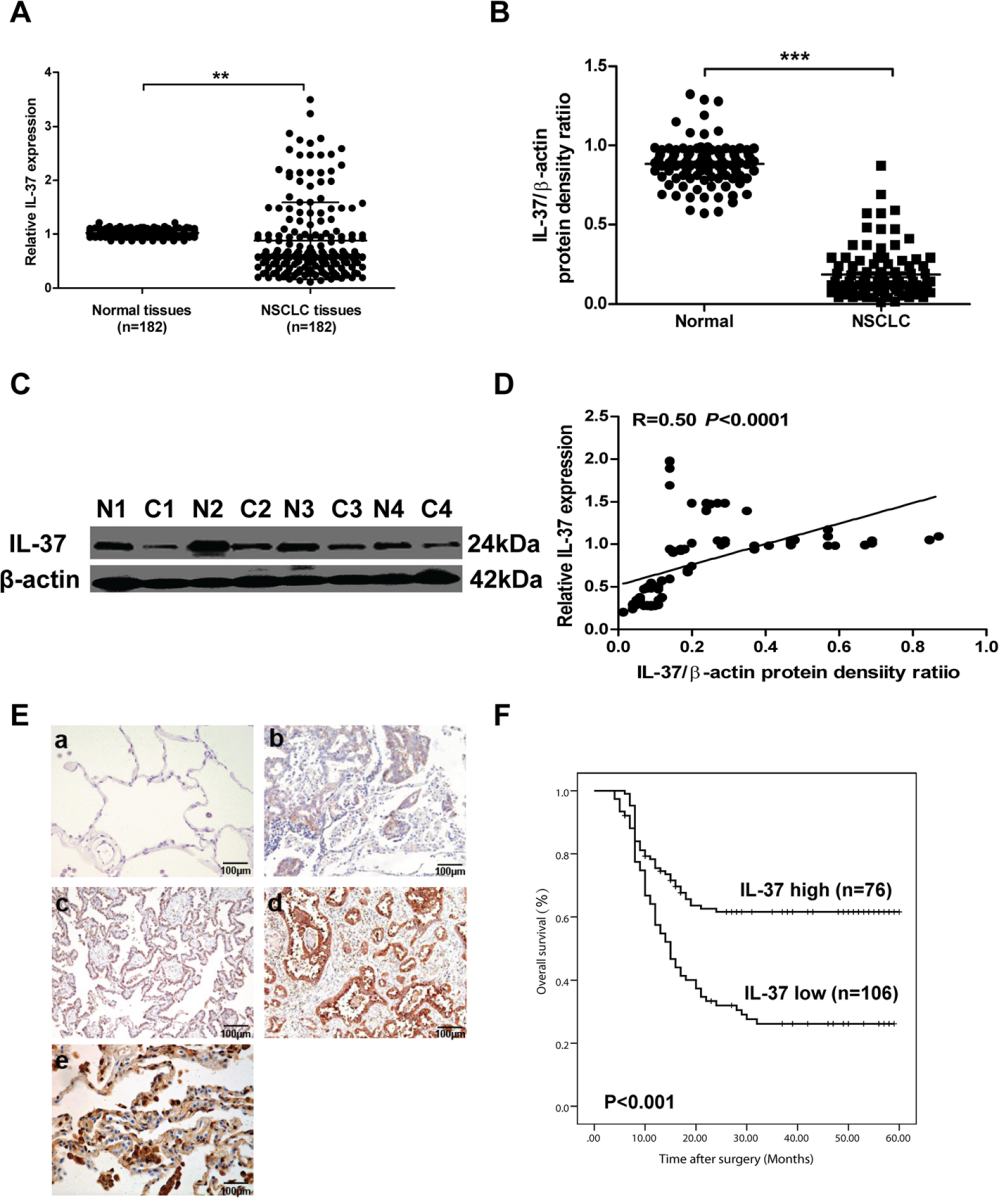
Decreased IL-37 expression is associated with poor prognosis in NSCLC patients. **a** The expression of IL-37 in 182 paired of NSCLC samples and their corresponding normal lung tissues was detected using qRT-PCR. **b** Western blot analysis of IL-37 expression in NSCLC samples (*n* = 97). Depicted are 4 individual pairs of NSCLC samples **c**. **d** IL-37 mRNA levels were positively correlated with its expression on protein levels. Each symbol represents an individual patient. The correlations were evaluated with Spearman’s non-parametric test. **e** Immunohistochemistry staining of human NSCLC sections using anti-IL-37 antibody (*n* = 182). **a** negative staining, and **e** strong staining in normal lung tissue; **b** weak (+), **c** moderate (++), **d** strong (+++) cytoplasmic staining in NSCLC tissues. Bar = 100 μm. **f** Kaplan-Meier curves of survival differences among NSCLC patients with high IL-37 expression (*n* = 76) and low IL-37 expression (*n* = 106) after surgery. *P* values were determined by the log-rank test. Data shown are mean ± SD. ^**^ *P* < 0.01, ^***^ *P* < 0.001

It has been reported that IL-37 mRNA expression has been found in diverse human tissues, including lung [3]. We first analyzed IL-37 expression in 182 NSCLC specimens. As shown in Fig.1a, mRNA levels of IL-37 in cancer tissues were lower when compared with corresponding normal tissues. Consistent with the mRNA data, we also found lower protein levels of IL-37 in NSCLC tissues than corresponding normal tissues (Fig. 1b and c), and IL-37 mRNA levels were positively correlated with its expression on protein levels (Fig. 1d). Moreover, immunostaining showed that the presence of IL-37-positive cells was mainly observed in the normal lung tissues, and was restricted to the cytoplasm. IL-37 expression was significantly lower in NSCLC tissues compared with normal lung tissues (Fig. 1e). Specifically, 76 of 182 tumor tissues (41.7 %) and 106 of 182 normal tissues (58.2 %) had a high level of IL-37 expression (*P* < 0.01). Furthermore, NSCLC patients with lower intratumoral IL-37 expression had significantly poorer OS than those with high IL-37 expression (Fig. 1f, *P <* 0.001, long rank test).

We then analyzed the relationship between the intratumoral IL-37 expression in NSCLC tissues and clinical characteristics. As shown in Table 1, there was no significant correlation of IL-37 expression to age (*P* = 0.670), gender (*P* = 0.343), histological type (*P* = 0.223), smoking status (*P* = 0.812) and differentiation (*P* = 0.914). However, low IL-37 expression was significantly correlated with higher tumor status (*P* = 0.017) and advanced TNM stage (I-II versus III, *P* = 0.000). In addition, univariate analysis showed that intratumoral IL-37 expression was prognostic factors for OS (Table 2). Multivariate Cox regression analysis revealed that intratumoral IL-37 expression was an independent prognostic factors for OS (hazard ratio [HR] =2.047; 95 % confidence interval [CI], 1.211-5.145; *P* = 0.011; Table 2). Taken together, these results suggested that the decrease in intratumoral IL-37 expression was associated with NSCLC progression and might be served as an independent predictor of poor survival.

**Table 2:** Univariate and multivariate analyses of factors associated with overall survival of NSCLC patients

Factors	Univariate	Multivariate		
	*P*	Hazard ratio	95 % CI	*P*
Age, years (≤60 *vs* >60)	0.924			
Gender (male *vs* female)	0.427			
Histological type (ADC *vs* non-ADC)	0.134			
Smoking status (smoker *vs* nonsmoker)	0.115			
Differentiation (well-moderate *vs* poor)	0.369			
Tumor status (T1-T2 *vs* T3-T4)	0.019	1.248	0.749-2.687	0.034
TNM stage (I-II *vs* III)	0.007	1.894	0.948-4.679	0.015
IL-37 expression (low *vs* high)	0.003	2.047	1.211-5.145	0.011

### Overexpression of IL-37 suppresses NSCLC tumorigenesis in vivo

**Fig. 2:**
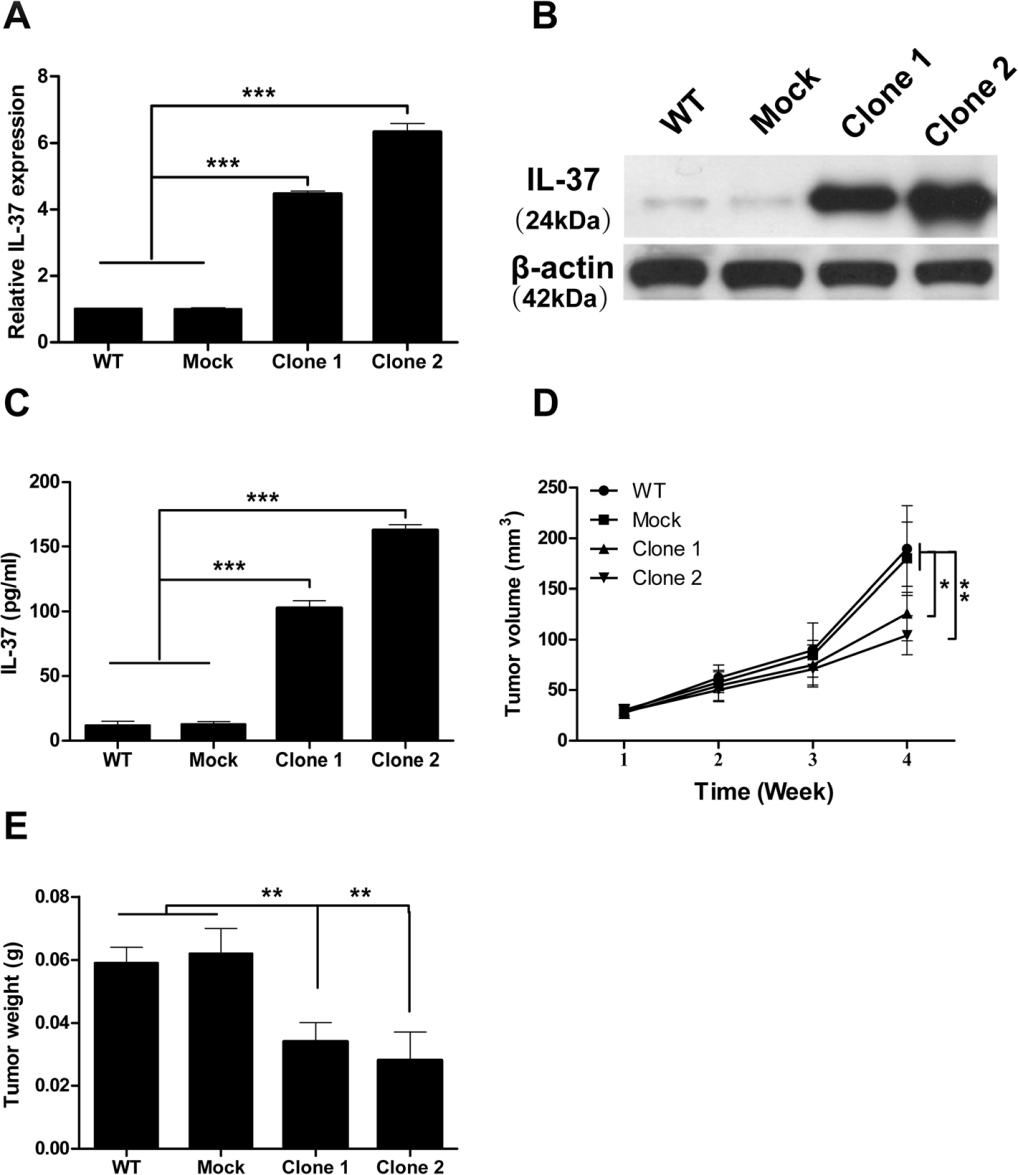
Overexpression of IL-37 suppresses NSCLC tumorigenesis in vivo*.* **a** qRT-PCR analysis of IL-37 mRNA expression from wild-type (WT), mock- transfected, and IL-37 transfected (clone 1 and 2) H1299 cells. **b** Western blot analysis of protein lysates from the cells. **c** ELISA analysis of IL-37 in supernatants of the cells. **d** Tumor volume during four weeks after the transplantation (*n* = 10 per group). **e** Tumor weights on week four after transplantation. Data shown are mean ± SD from three independent experiments. ^*^ *P* < 0.05, ^**^ *P* < 0.01, ^***^ *P* < 0.001

To investigate the role of IL-37 in tumorigenesis, we stably transfected lung cancer cell line H1299 cells with IL-37 cDNA and empty vector as control (mock transfectant). qRT-PCR analysis of mRNA levels (Fig. 2a) and western blot analysis of the cellular proteins levels (Fig. 2b) both showed abundant IL-37 expression in the IL-37-transfected monoclonal H1299 cells, while undetectable IL-37 in mock-transfected and wild-type H1299 cells. ELISA analysis of cultural supernatants also showed the expression of IL-37 was remarkably increased in IL-37 transfected H1299 cells (Fig. 2c).

We then evaluated the effect of IL-37 on cell growth, cell cycle and cell apoptosis using IL-37-transfected, mock-transfected and wild-type H1299 cells in vitro. FACS analysis showed no significant difference in cell apoptosis and cell cycle among the four groups (Additional file 1: Fig. S1A and B). Time course CCK-8 assay showed identical cell growth among the four groups (Additional file 1: Fig. S1C). We further confirmed these results using another lung cancer cell line A549 (Additional file 2: Fig. S2A-C). These results suggested that IL-37 may not directly affect apoptosis and proliferation of lung cancer cells in vitro.

We next used a xenograft NSCLC model to elucidate the effects of IL-37 on NSCLC progression in vivo. The results showed that IL-37-transfected H1299 cells grew significantly slower than mock-transfected and wild-type cells when transplanted in nude mice (Fig. 2d). 4 weeks after transplantation, the mean tumor weights of 0.034 g, 0.028 g were found in two different IL-37-transfected tumors, and 0.059 g, 0.062 g in wild type and mock-transfected tumors (Fig. 2e). These results suggested that IL-37 appears to inhibit the tumor growth of NSCLC in vivo.

### IL-37 negatively regulates tumor angiogenesis

IL-37 is proved to be a natural suppressor of innate inflammatory and immune response. Previous study also showed that IL-37 mediated anti-tumor immune responses through recruiting NK cells to tumor microenvironment in HCC. So we first analyzed cell frequencies of several immune cell subsets in H1299 transplanted tumors. We found similar cell populations based upon CD11c, CD11b, Gr-1 and DX5 stainings in IL-37- and mock-transfected tumors (Additional file 1: Fig. S1D), suggesting that IL-37 did not affect the anti-tumor immune responses in vivo.

**Fig. 3:**
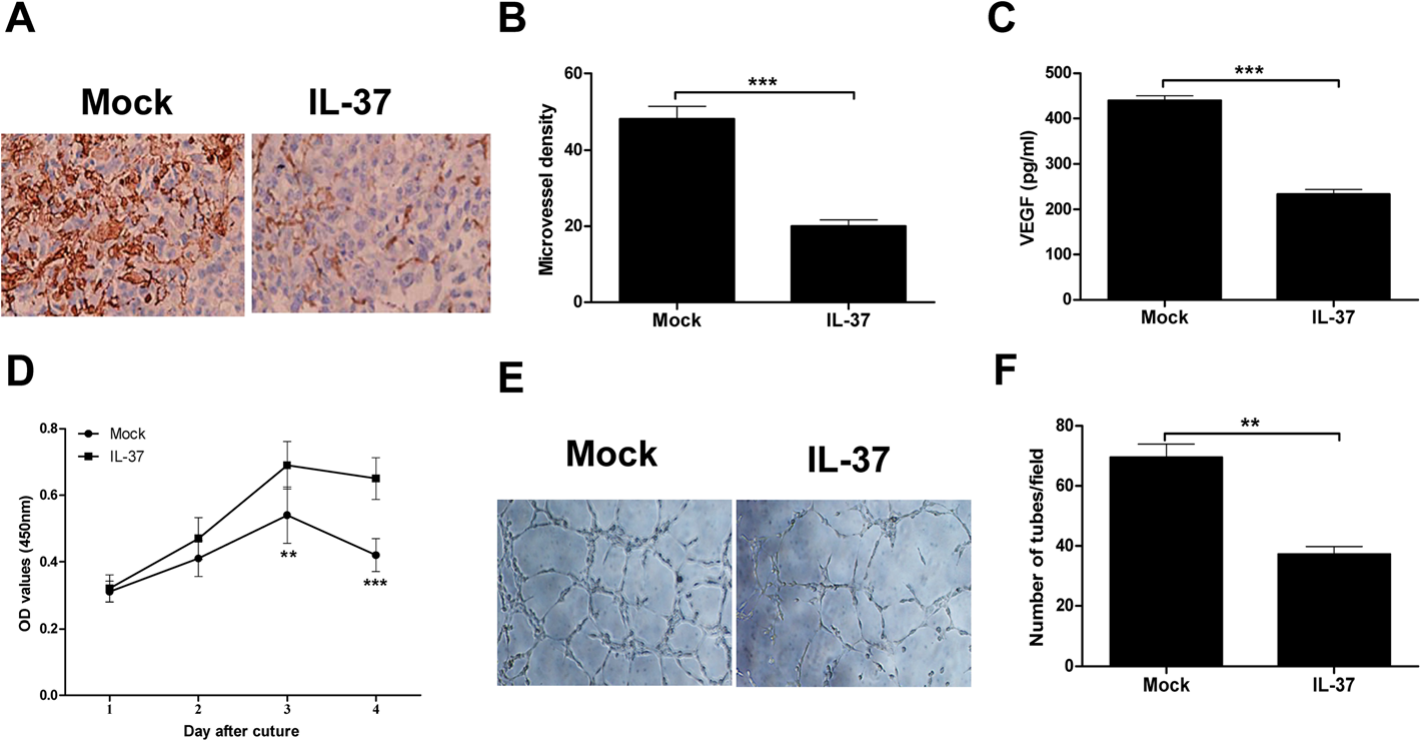
IL-37 negatively regulates tumor angiogenesis. **a** Immunohistochemistry staining of transplanted tumors of IL-37- and mock-transfected H1299 cells using anti-CD34 antibody. Representative pictures for two groups are shown. **b** The tumor microvessel density in two groups was counted. **c** ELISA analysis of VEGF levels in the cultural supernatants of the IL-37 transfected and mock-transfected H1299 cells. **d** CCK-8 analysis of HUVEC cells cultured in conditioned medium from supernatants of IL-37 or mock transfected H1299 cells. **e** The effect of IL-37 on the capillary structure formation was measured using matrigel tubule formation assay. Representative pictures for two groups are shown. **f** The number of formed capillary tubes in two groups were counted. Data shown are mean ± SD from three independent experiments. ^**^ *P* < 0.01, ^***^ *P* < 0.001

Tumor angiogenesis is essential for the development and progression of malignant tumors, including NSCLC. Anti-angiogenic strategies like bevacizumab have been developed into standard treatment options in NSCLC [30]. No studies have shown the correlation of IL-37 with tumor angiogenesis in NSCLC. Thus we examine the tumor angiogenesis in the nude mice tumor model. As shown in Fig. 3a, the CD34 expression in the IL-37-transfected tumors was weaker or less, compared with the mock-transfected tumors. In addition, the density of microvessel counted in the IL-37-transfected tumors was significantly less than that in the mock-transfected tumors (Fig. 3b). These results implied that IL-37 may suppress tumor growth by inhibiting tumor angiogenesis in vivo.

To address the direct antiangiogenic effect of IL-37, we first examined the VEGF levels in the cultural supernatants of the IL-37 transfected and mock-transfected H1299 cells. As shown in Fig. 3c, the amounts of VEGF in IL-37-transfected H1299 cells was significant less than that in mock-transfected H1299 cells. Next we treated HUVEC cells with conditional medium from supernatants of IL-37 or mock transfected H1299 cells. Results showed that IL-37 treatment significantly inhibited HUVEC growth in a time dependent manner (Fig. 3d). Additionally, matrigel tubule formation assay was used as an in vitro model to study the effect of IL-37 on the capillary structure formation. As shown in Fig. 3e, IL-37-treated cells had reduced branching points, tubule number and length, and the number of HUVEC capillary structures was significantly decreased by IL-37 treatment (Fig. 3f). Taken together, these results indicated that IL-37 may directly regulate angiogenesis in vitro and in vivo.

### Decreased IL-37 expression is associated with high MVD in NSCLC patients

**Fig. 4:**
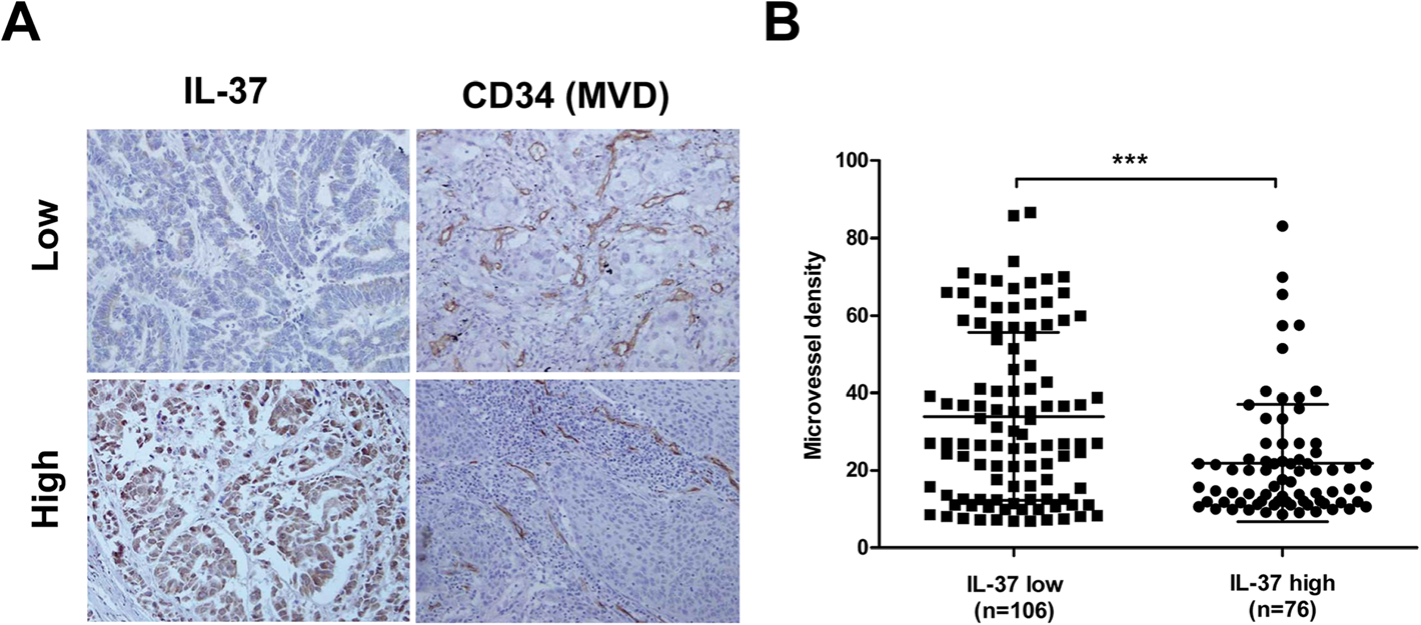
Decreased IL-37 expression is associated with high MVD in NSCLC patients. **a** Representative immunohistochemical staining of IL-37 and CD34 in the same surgical specimens from patients with high or low expression of IL-37. **b** MVD in relation to IL-37 expression status was analyzed. Data shown are mean ± SD. ^***^ *P* < 0.001

We next analyzed the correlation between MVD and the intratumoral IL-37 expression in human NSCLC samples. Intratumoral MVD was quantified by counting CD34-positive endothelial cells in the same series of lung cancer tissues, and the staining intensity of MVD ranged broadly from 7 to 86 microvessels/200× magnification fields (Fig. 4a). In addition, The MVD was significantly higher in IL-37-low expression tumors (34 ± 21) than those in IL-37-high expression tumors (21 ± 15) (Fig. 4b).

## Discussion

IL-37 has been identified as a natural suppressor of innate inflammatory and immune responses[8]. It is highly expressed in inflammatory tissues to inhibit the excessive inflammatory response. Recent studies indicate that IL-37 plays a protective role in tumor progression in mouse fibrosarcoma, human HCC and CC [7, 26, 27]. However, there is no information about whether IL-37 influences the pathogenesis of NSCLC development, progression, and prognosis.

In the current study, we first investigated the expression pattern of IL-37 protein and its clinical significance in NSCLC patients. Using immunohistochemical staining, we found that IL-37 was expressed in non-tumor tissues, which was down-regulated in lung cancer tissues. Zhao et al. [26] reported that the IL-37 expression level was significantly negatively associated with tumor size in HCC, indicating that IL-37 might inhibit tumor growth in the tumor microenvironment. Here we also found that low intratumoral IL-37 expression was correlated with advanced TNM stage and poor OS, suggests that IL-37 may play an inhibitory role in the development of NSCLC.

To confirm our findings of clinical, we constructed lung cancer cell line H1299 that overexpressed IL-37; we found that IL-37 did not directly inhibit cell growth of lung cancer cells in vitro. However, over-expression of IL-37 inhibited the in vivo growth of H1299 cells, indicating that IL-37 repressed NSCLC tumorigenesis in vivo. However, contrary to our findings, Wang et al. [27] reported that over-expression IL-37 in cervical cancer cell lines suppressed cell proliferation and invasion. Since cervical cancer is related to human papillomavirus (HPV) infection and the chronic inflammation of the body [31, 32], and chronic inflammation is closed connected with tumorigenesis [33], IL-37 may play diverse role in different tumors.

What is the mechanism underlying the inhibition of NSCLC tumorigenesis by IL-37? Since IL-37 did not affect cell proliferation and apoptosis, it is unlikely that IL-37 directly affects in vivo proliferation of the transplanted lung tumors. IL-37 is a natural suppressor of immune responses. Previous study also showed that IL-37 mediated anti-tumor immune responses through recruiting NK cells to tumor microenvironment in HCC. However, we found no change in TILs frequencies in tumors over-expressing IL-37, suggesting that IL-37 did not affect the anti-tumor immune responses in vivo. In the meantime, we found IL-37-over-expressing tumors had decreased CD34 level, which suggested an inhibited tumor angiogenesis. And we all known that tumor angiogenesis is essential for the development and progression of malignant tumors, including NSCLC [34]. On the other hand, we found that IL-37-transfected H1299 cells had low levels of VEGF, and IL-37 treatment significantly inhibited HUVEC growth and tubule formation. Together, our findings suggest that IL-37 suppresses lung cancer development possibly through inhibiting tumor angiogenesis.

In support of cell-based findings, we also found a significant negative correlation between intratumoral IL-37 expression and tumor angiogenesis measured as CD34-determined intratumoral MVD in human NSCLC samples, suggesting an inhibitory role of IL-37 in the tumor angiogenesis. However, tumor angiogenesis is an extremely complex process involving multistep process and diffusible factors. Thus, the exact molecular mechanisms by which IL-37 contributes to anti-angiogenic potential of tumors are far from clear and still need to be further elucidated.

## Conclusion

In conclusion, we provide evidence for the first time that IL-37 was decreased in human NSCLC and inhibits lung tumorigenesis in vivo, possibly by inhibiting tumor angiogenesis. Our findings suggest that IL-37 may have clinical potential not only as a promising prognostic predictor to identify individuals with poor prognostic potential, but also as a novel therapeutic target in antiangiogenesis for NSCLC patients. Further investigations are necessary to clarify and understand the mechanisms of IL-37 in angiogenesis in NSCLC.

## Additional files

Additional file 1: Figure S1. IL-37 does not affect cell growth, apoptosis and cell cycle in vitro and immune cell subsets in vivo. Cell apoptosis (A) and cell cycle (B) of IL-37-transfected, mock-transfected and wild-type H1299 cells were analyzed by FACS. (C) Cell growth of IL-37-transfected, mock-transfected and wild-type H1299 cells was analyzed by CCK-8 assay. (D) The immune cell subsets in transplanted tumors of IL-37-transfected and mock-transfected H1299 cells were measured by FACS. Data shown are mean ± SD from three independent experiments. (TIF 972 kb) Additional file 2: Figure S2. IL-37 does not affect A549 cell growth, apoptosis and cell cycle in vitro. Cell apoptosis (A) and cell cycle (B) of IL-37-transfected, mock-transfected and wild-type A549 cells were analyzed by FACS. (C) Cell growth of IL-37-transfected, mock-transfected and wild-type A549 cells was analyzed by CCK-8 assay. Data shown are mean ± SD from three independent experiments. (TIF 669 kb)

## Abbreviations

NSCLC: Non-small cell lung cancer
MVD: Microvessel density
HUVEC: Human umbilical vein endothelial cells
qRT-PCR: Quantitative reverse transcription PCR
