# Supplementary figures and images for: Interleukin-37 suppresses tumor growth through inhibition of angiogenesis in non-small cell lung cancer
Source: J Exp Clin Cancer Res. 2016 Jan 20;35:13. doi: 10.1186/s13046-016-0293-3 (PMC4721009; doi:10.1186/s13046-016-0293-3)

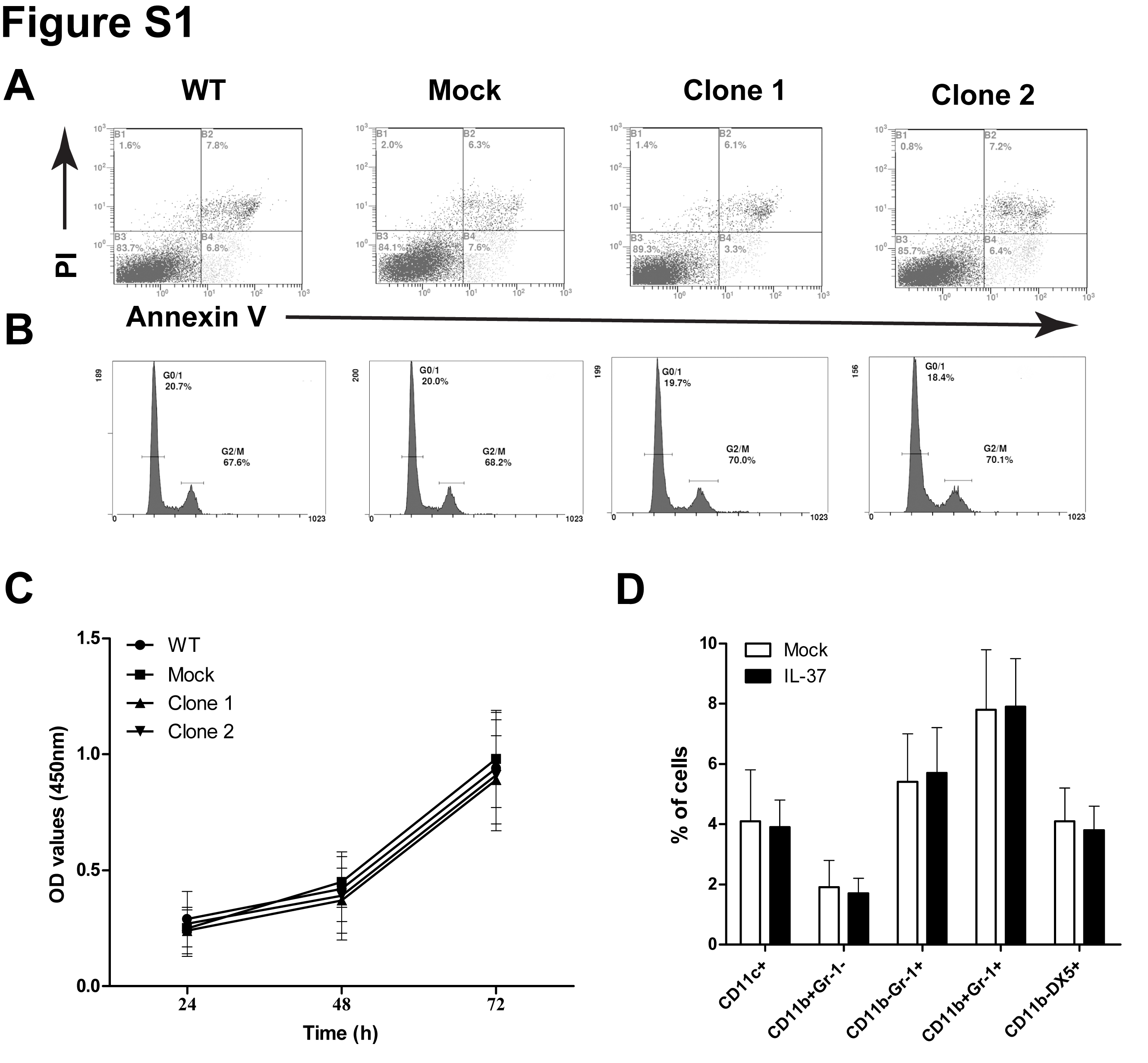

Supplement: Additional file 1: Figure S1. — IL-37 does not affect cell growth, apoptosis and cell cycle in vitro and immune cell subsets in vivo. Cell apoptosis (A) and cell cycle (B) of IL-37-transfected, mock-transfected and wild-type H1299 cells were analyzed by FACS. (C) Cell growth of IL-37-transfected, mock-transfected and wild-type H1299 cells was analyzed by CCK-8 assay. (D) The immune cell subsets in transplanted tumors of IL-37-transfected and mock-transfected H1299 cells were measured by FACS. Data shown are mean ± SD from three independent experiments. (TIF 972 kb) [file 13046_2016_293_MOESM1_ESM.tif]

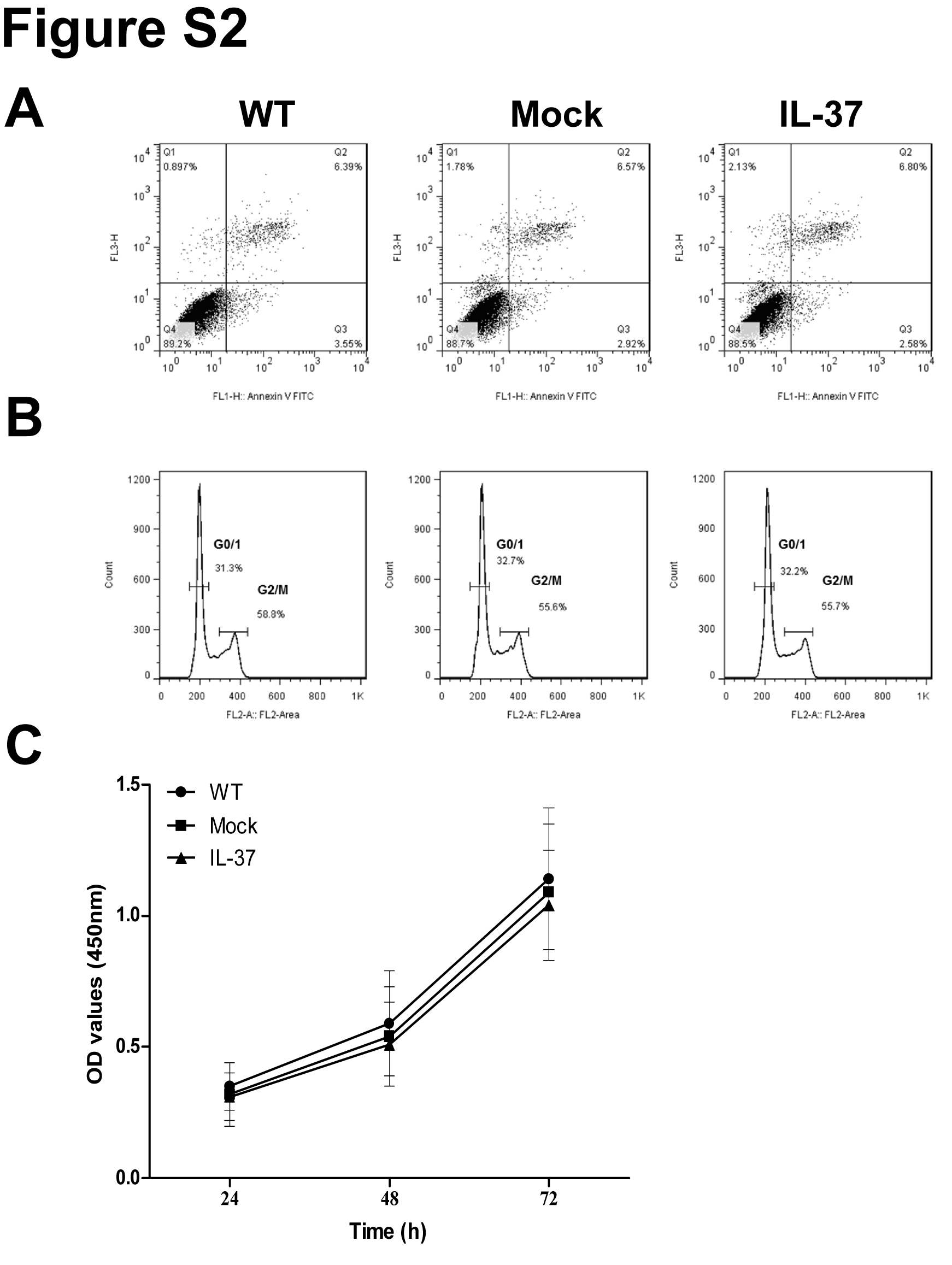

Supplement: Additional file 2: Figure S2. — IL-37 does not affect A549 cell growth, apoptosis and cell cycle in vitro. Cell apoptosis (A) and cell cycle (B) of IL-37-transfected, mock-transfected and wild-type A549 cells were analyzed by FACS. (C) Cell growth of IL-37-transfected, mock-transfected and wild-type A549 cells was analyzed by CCK-8 assay. Data shown are mean ± SD from three independent experiments. (TIF 669 kb) [file 13046_2016_293_MOESM2_ESM.tif]
